# Reactive Cutaneous Capillary Endothelial Proliferation in Esophageal Squamous Cell Carcinoma Patients Treated With Camrelizumab‐Based Therapy: A Pooled Analysis of Two Phase 3 Trials

**DOI:** 10.1002/mco2.70917

**Published:** 2026-08-09

**Authors:** Jia Liu, Ye He, Mingming He, Fan Chen, Zhiguo Hou, Zhaoqing Zheng, Chaoyu Lin, Aichun Chen, Ruihua Xu, Huiyan Luo, ESCORT‐1st Study Group, ESCORT Study Group

**Affiliations:** ^1^ Department of Medical Oncology Sun Yat‐sen University Cancer Center Guangzhou China; ^2^ Department of Medical Affairs Jiangsu Hengrui Pharmaceuticals Co., Ltd. Jiangsu China

**Keywords:** camrelizumab, esophageal squamous cell carcinoma, reactive cutaneous capillary endothelial proliferation

## Abstract

Reactive cutaneous capillary endothelial proliferation (RCCEP) is the most common immune‐related adverse event associated with camrelizumab and has been linked to favorable outcomes. We conducted a pooled analysis of two phase III randomized trials, ESCORT (NCT03099382) and ESCORT‐1st (NCT03691090), to evaluate the clinical characteristics and prognostic relevance of RCCEP in patients with advanced esophageal squamous cell carcinoma (ESCC) receiving camrelizumab‐based therapy. A total of 526 patients treated with camrelizumab, either alone or in combination with chemotherapy, were included. Objective response rate (ORR), progression‐free survival (PFS), and overall survival (OS) were compared according to RCCEP status. Inverse probability of treatment weighting (IPTW) was applied to adjust for baseline imbalances, and a 2‐month landmark analysis was performed to reduce lead‐time bias. Overall, 420 patients (79.8%) developed RCCEP, mostly grade 1–2 (99.0%), with no RCCEP‐related treatment discontinuations. The median onset was 1.18 months, and the duration was 3.12 months. RCCEP‐positive patients showed a higher ORR (52.4% vs. 26.4%), longer PFS (5.55 vs. 1.94 months; HR = 0.45), and OS (13.40 vs. 6.11 months; HR = 0.47), consistent after landmark analysis. These findings suggested that RCCEP was frequent yet manageable and may serve as a potential on‐treatment biomarker of camrelizumab benefit in ESCC.

## Graphical abstract

Camrelizumab‐induced reactive cutaneous capillary endothelial proliferation (RCCEP) was frequent yet manageable in patients with advanced esophageal squamous cell carcinoma and correlated with improved response and survival, suggesting RCCEP as a potential on‐treatment biomarker of clinical benefit.

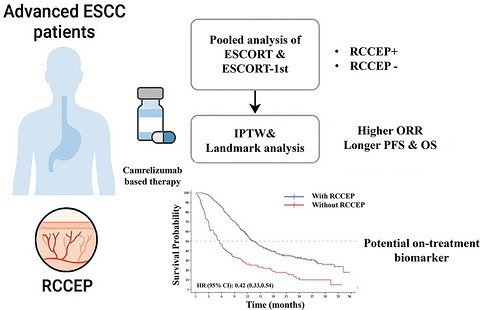

## 1 Introduction

Esophageal cancer is the seventh leading cause of cancer‐related mortality worldwide, and esophageal squamous cell carcinoma (ESCC) is the predominant subtype in Asia [1, 2]. Despite advancements in multimodal treatment approaches, outcomes for advanced or metastatic ESCC remain poor [3]. The introduction of immune checkpoint inhibitors (ICIs), particularly monoclonal antibodies targeting programmed death‐1 (PD‐1) and its ligand PD‐L1, has transformed the treatment landscape for locally advanced and metastatic cases [4]. Among these, camrelizumab, a humanized anti‐PD‐1 monoclonal antibody, has demonstrated significant clinical benefits in ESCC, as evidenced by the phase III ESCORT [5] and ESCORT‐1st [6] trials, leading to its regulatory approval as a first‐line and second‐line treatment option.

With the expanding application of ICI‐based treatments, clinical awareness of immune‐related adverse events (irAEs) has become increasingly important, drawing attention to their prominence [7]. These irAEs commonly affect multiple tissues and organ systems, including the skin, respiratory, gastrointestinal, and endocrine systems [8], necessitating careful monitoring and management. Among these, reactive cutaneous capillary endothelial proliferation (RCCEP) has emerged as a distinct and predominant irAE observed in patients receiving camrelizumab. RCCEP is characterized by abnormal proliferation of capillary endothelial cells, distinguishing it from the lichenoid dermatitis associated with nivolumab and pembrolizumab [9, 10]. As the most common dermatologic toxicity linked to camrelizumab therapy [11, 12, 13], RCCEP is typically mild and self‐limiting, with a low incidence of treatment discontinuation [14]. However, its visually striking clinical presentation raises concerns regarding treatment adherence, primarily manifesting as a cosmetic burden, underscoring the need for standardized management strategies.

An increasing body of evidence suggested that the onset of irAEs might serve as a predictive marker of treatment response to PD‐1/PD‐L1 inhibitors across various malignancies, including melanoma and non‐small cell lung cancer [15, 16]. Most studies reported that patients who develop irAEs experience significantly improved progression‐free survival (PFS), overall survival (OS), and objective response rates (ORR) compared to those without irAEs. Similarly, the occurrence of RCCEP has been reported to correlate with the efficacy of camrelizumab in patients with advanced hepatocellular carcinoma (HCC) and recurrent/metastatic head and neck squamous cell carcinoma [14, 17]. Further pooled analyses evaluating both camrelizumab monotherapy and combination regimens have reinforced RCCEP as a potential on‐treatment biomarker of response and prognosis in multiple cancer types [18]. However, few studies have investigated this relationship in patients with ESCC.

In ESCORT and ESCORT‐1st, RCCEP was consistently observed in approximately 80% of patients receiving camrelizumab‐based treatment. Understanding the potential prognostic implications of RCCEP could offer valuable insights into ESCC treatment optimization and may also improve patient adherence by reinforcing its association with therapeutic benefit. In this post hoc exploratory analysis, we systematically characterized the clinical features of RCCEP in patients with ESCC and assessed its correlation with survival outcomes, using pooled data from ESCORT and ESCORT‐1st.

## 2 Results

### 2.1 Patient Characteristics and Clinical Features of RCCEP

Overall, 526 patients receiving either camrelizumab monotherapy or camrelizumab in combination with chemotherapy were included in the analyses of demographic and baseline characteristics. Of these patients, 420 (79.8%) developed RCCEP and 106 did not. The baseline characteristics are shown in Table 1. To minimize confounding factors, IPTW method was applied. After weighting, the weighted number of patients was 420.19 with RCCEP and 105.27 without RCCEP, both with a median (IQR) age of 61.0 years (55.0–65.0).

**TABLE 1:** Baseline characteristics before and after IPTW.

Characteristics	Before IPTW				After IPTW		
RCCEP	With (*N* = 420)	Without (*N* = 106)		SMD	With (*N* = 420.19^a^)	Without (*N* = 105.27^a^)	SMD
Age (years), median (IQR)	61.0 (55.0–65.0)		61.0 (56.0–65.0)	0.002	61.0 (55.0–65.0)	61.0 (55.0–65.0)	0.020
Sex				−0.065			−0.079
Male	372 (88.6)		96 (90.6)		372.3 (88.6)	95.8 (91.0)	
Female	48 (11.4)		10 (9.4)		47.9 (11.4)	9.5 (9.0)	
ECOG PS				0.196			0.041
0	100 (23.8)		17 (16.0)		93.1 (22.2)	21.6 (20.5)	
1	320 (76.2)		89 (84.0)		327.1 (77.8)	83.7 (79.5)	
Therapy line				−0.001			0.046
Second	182 (43.3)		46 (43.4)		181.7 (43.2)	43.1 (41.0)	
First	238 (56.7)		60 (56.6)		238.5 (56.8)	62.1 (59.0)	
Liver metastases				0.295			0.019
No	332 (79.0)		70 (66.0)		320.8 (76.3)	79.5 (75.5)	
Yes	88 (21.0)		36 (34.0)		99.4 (23.7)	25.8 (24.5)	
PD‐L1 expression (TPS)^b^				−0.095			−0.062
< 1%	200 (47.6)		55 (51.9)		200.6 (47.7)	53.1 (50.5)	
≥1%	211 (50.2)		48 (45.3)		210.6 (50.1)	49.3 (46.9)	

*Note*: Data are *n* (%) unless otherwise indicated.

^a^ Values were presented as weighted ESS after IPTW adjustment.

^b^ PD‐L1 TPS data were available only in evaluable patients with available PD‐L1 expression assessment.

Abbreviations: ECOG PS, Eastern Cooperative Oncology Group performance status; ESS, effective sample sizes; IPTW, inverse probability of treatment weighting; IQR, interquartile range; RCCEP, reactive cutaneous capillary endothelial proliferation; SMD, standardized mean difference; TPS, tumor proportion score.

Of the 420 patients with RCCEP, the majority experienced grade 1 (356 patients [84.8%]) or grade 2 (60 patients [14.3%]) severity, while grade 3 was rare (4 patients [1.0%]). No grade 4 or 5 RCCEP events were observed. RCCEP occurred a median of 1.0 time (IQR: 1.0–1.0). The median time to first RCCEP onset was 1.18 months (IQR: 0.79–1.87), and the median duration of RCCEP lesions was 3.12 months (range, 0.07–15.01). No patients required treatment interruption or discontinuation due to RCCEP, and no systemic or invasive interventions specifically targeting RCCEP were reported in this cohort.

### 2.2 Correlation Between RCCEP and the Efficacy of Camrelizumab

As shown in Table 2, after IPTW, 34.4 of 420.19 (8.19%) patients with RCCEP achieved complete response (CR), while 184.2 (43.83%) achieved partial response (PR). In contrast, among the 105.27 patients without RCCEP, only 4.3 (4.07%) achieved CR and 25.5 (24.25%) achieved PR. A significant association between RCCEP occurrence and a higher ORR was observed both before and after IPTW. After IPTW, ORR was 52.03% (95% CI, 47.25–56.80) in patients with RCCEP compared to 28.32% (95% CI, 19.71–36.93) in those without RCCEP.

**TABLE 2:** Radiological assessment of short‐term efficacy.

	Before IPTW		After IPTW	
RCCEP	With (*N* = 420)	Without (*N* = 106)	With (*N* = 420.19)	Without (*N* = 105.27)
CR	35 (8.3)	4 (3.8)	34.4 (8.19)	4.3 (4.07)
PR	185 (44.0)	24 (22.6)	184.2 (43.83)	25.5 (24.25)
SD	106 (25.2)	16 (15.1)	104.9 (24.96)	16.4 (15.57)
PD	86 (20.5)	38 (35.8)	88.4 (21.03)	36.3 (34.49)
NE ORR (CR+PR)	8 (1.9) 220 (52.4)	24 (22.6) 28 (26.4)	8.3 (1.99) 218.6 (52.03)	22.8 (21.62) 29.8 (28.32)
95% CI	(47.5–57.2)	(18.3–35.9)	(47.25–56.80)	(19.71–36.93)

*Note*: Data are *n* (%).

Abbreviations: CR, complete response; NE, not evaluated; ORR, overall response rate; PD, progressive disease; PR, partial response; SD, stable disease.

**FIGURE 1:**
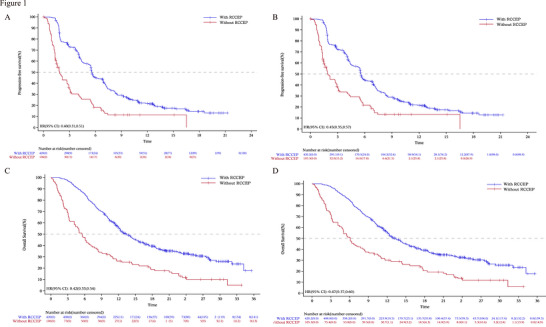
Kaplan–Meier survival curves for PFS (A, B) and OS (C, D) in patients with and without RCCEP, shown before IPTW (A, C) and after IPTW (B, D). IPTW, inverse probability of treatment weighting; OS, overall survival; PFS, progression‐free survival; RCCEP, reactive cutaneous capillary endothelial proliferation.

In terms of long‐term efficacy analysis, significant associations between RCCEP occurrence and prolonged PFS and OS were observed. The Kaplan–Meier curves for PFS and OS were shown in Figure 1. After weighting, median PFS reached 5.55 (95% CI, 5.45–5.85) months versus 1.94 (95% CI, 1.48–2.92) months in patients with RCCEP versus those without RCCEP (HR, 0.45 [95% CI,0.35–0.57], log‐rank test *p* < 0.001, Figure 1B). The median OS was 13.40 months (95% CI, 12.09–14.72) in patients with RCCEP and 6.11 months (95% CI, 4.73–8.11) in patients without RCCEP (HR, 0.47 [95% CI, 0.37–0.60], log‐rank test *p* < 0.001, Figure 1D). Additional analyses evaluating the associations between RCCEP occurrence and survival outcomes stratified by treatment line (first‐line and second‐line) were presented in Table S1.

### 2.3 Landmark Analyses

**FIGURE 2:**
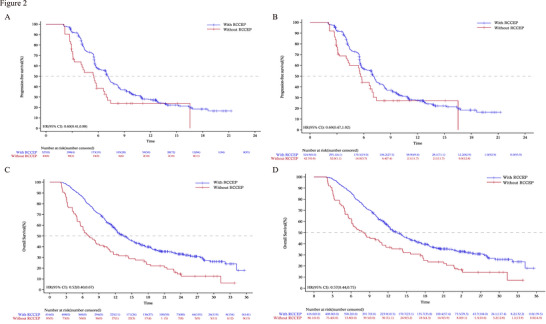
Landmark analysis of survival between patients with and without RCCEP. Kaplan–Meier survival curves with 2‐month landmark analysis for PFS (A, B) and OS (C, D) in patients with and without RCCEP, shown before IPTW (A, C) and after IPTW (B, D). IPTW, inverse probability of treatment weighting; OS, overall survival; PFS, progression‐free survival; RCCEP, reactive cutaneous capillary endothelial proliferation.

Using the median time to first RCCEP onset as the cutoff, a 2‐month landmark survival analysis was further performed (Table 3). Before IPTW, 369 and 501 patients were included in the PFS and OS landmark analyses, respectively (Figure S1). The results further supported the association between RCCEP occurrence and improved survival outcomes, with both PFS and OS being longer in patients with RCCEP than in those without (Figure 2). After IPTW, landmark analyses demonstrated that patients with RCCEP had a lower risk of progression or death compared to those without RCCEP, with an HR of 0.69 (95% CI, 0.47–1.02; log‐rank test, *p* = 0.075) for PFS (Figure 2B) and an HR of 0.57 (95% CI, 0.44–0.75; log‐rank test, *p* < 0.001) for OS (Figure 2D). These findings suggested a generally consistent trend toward favorable survival outcomes in patients with RCCEP, particularly for OS.

**TABLE 3:** Two‐month landmark analysis for PFS and OS.

	Before IPTW		After IPTW	
Progression‐free survival (PFS)	With RCCEP (*N* = 327)	Without RCCEP (*N* = 42)	With RCCEP (*N* = 324.9)	Without RCCEP (*N* = 42.7)
Median PFS, months (95% CI)	7.13 (6.64–7.43)	5.49 (3.12–6.74)	7.06 (6.34–7.43)	5.65 (4.04–7.06)
Hazard ratio (95% CI)	0.60 (0.41–0.88)		0.69 (0.47–1.02)	
Log‐rank test	*p* = 0.007		*p* = 0.075	
Overall survival (OS)	With RCCEP (*N* = 416)	Without RCCEP (*N* = 85)	With RCCEP (*N* = 416.0)	Without RCCEP (*N* = 86.1)
Median OS, months (95% CI)	13.44 (12.32–15.05)	7.13 (5.55–10.48)	13.44 (12.25– 15.05)	8.11 (6.08–11.04)
Hazard ratio (95% CI)	0.52 (0.40, 0.67)		0.57 (0.44, 0.75)	
Log‐rank test	*p* < 0.001		*p* < 0.001	

## 3 Discussion

In this post hoc exploratory pooled analysis of the phase 3 ESCORT and ESCORT‐1st randomized clinical trials, we investigated the incidence and clinical significance of RCCEP in patients with ESCC receiving camrelizumab‐based therapy. Our findings revealed a high RCCEP incidence (79.8%), with the majority of cases being mild (grade 1–2 in 99.0%), aligning with previous reports and reinforcing RCCEP as a characteristic irAE associated with camrelizumab. After IPTW, patients who developed RCCEP exhibited significantly improved short‐term response (ORR) and prolonged survival outcomes (PFS and OS). Additionally, to minimize potential bias caused by patients having to undergo treatment or living longer to experience irAEs, we performed a detailed landmark evaluation for both PFS and OS. The findings supported the hypothesis that the presence of RCCEP was associated with long‐term therapeutic advantages among patients treated with camrelizumab. However, given the *post hoc* and exploratory nature of the pooled analysis, these findings should be interpreted as hypothesis‐generating and warrant further validation in prospective studies.

Consistent with our findings in ESCC, RCCEP occurs at a relatively high frequency during camrelizumab monotherapy across multiple tumor types, although the incidence varies by disease context, ranging from approximately 66.8% in HCC to 67.2% in recurrent/metastatic head and neck squamous cell carcinoma (R/M HNSCC), and reaching up to 97.3% in classical Hodgkin lymphoma [14, 17, 19]. Notably, the favorable prognostic association of RCCEP appears to be observed across tumor types. In advanced HCC, patients who developed RCCEP achieved a significantly higher ORR (19.3% vs. 5.6%) and markedly prolonged OS (17.0 vs. 5.8 months; HR = 0.33) [14]. Similarly, in R/M HNSCC, RCCEP occurrence was associated with improved ORR (48.7% vs. 10.5%) and longer median OS (17.0 vs. 8.7 months) [17].

Given that irAEs arise from antigen‐specific immune activation and have been linked to improved clinical outcomes, a potential association between the two has been hypothesized [20]. Several studies in malignancies such as melanoma, non‐small cell lung cancer, and HCC have supported a correlation between irAEs and favorable treatment responses [21, 22, 23]. However, limited evidence—primarily from individual clinical studies, including subgroup analyses—has explored this relationship in patients with ESCC [24, 25]. In this study, we leveraged phase 3 randomized clinical trial data to provide rigorous validation, confirming the robustness of this association. Focusing on RCCEP, the most common irAE associated with camrelizumab, we found a strong correlation between RCCEP incidence and improved clinical outcomes. After IPTW in the landmark analysis, patients who developed RCCEP experienced a 43% reduction in the risk of death compared to those who did not, a result consistent with prior reports on RCCEP‐efficacy associations [14, 17, 18]. Furthermore, with a median onset time of 1.18 months (IQR: 0.79–1.87), RCCEP may serve as a potential on‐treatment indicator of therapeutic response, enabling clinicians to identify potential responders sooner and mitigate the risk of premature treatment discontinuation. Similar to other irAEs, the predictive potential of RCCEP warrants further exploration within a comprehensive biomarker framework, incorporating PD‐L1 expression, tumor mutational burden, and microsatellite instability [26, 27].

Unlike common cutaneous irAEs such as rash, pruritus, and vitiligo, which occur in 30%–40% of patients treated with other PD‐1/PD‐L1 inhibitors [28], RCCEP appears to be a distinct phenomenon likely driven by camrelizumab's unique immunomodulatory properties and downstream effects on vascular endothelial homeostasis. On one hand, camrelizumab‐mediated immune reactivation through CD4+/CD8+ T lymphocytes may promote the release of vascular endothelial growth factor A (VEGF‐A), thereby perturbing the balance between pro‐ and anti‐angiogenic signals and contributing to reactive endothelial changes in dermal capillaries [14, 29, 30]. From this perspective, RCCEP may represent an epiphenomenon of systemic immune activation. On the other hand, preclinical studies suggested that camrelizumab may interact with VEGF receptor 2 (VEGFR2) and frizzled class receptor 5 (FZD5), potentially contributing to vascular‐related cutaneous manifestations [31, 32]. This mechanistic insight aligns with clinical findings, which suggest the involvement of the VEGF signaling pathway in RCCEP pathogenesis. Notably, studies have demonstrated a significant reduction in RCCEP incidence when camrelizumab is co‐administered with apatinib, a VEGFR inhibitor, with rates decreasing from over 66.8% with camrelizumab alone to less than 21.9% when combined with apatinib [14, 33, 34]. Taken together, these findings suggest that RCCEP may arise from a complex interplay between immune activation and vascular endothelial responses. However, the current analysis lacks correlative biomarker data (e.g., circulating VEGF levels or endothelial activation markers), and future prospective studies integrating such translational endpoints are warranted to further elucidate the biological mechanisms underlying RCCEP.

Given its high incidence but limited impact on treatment tolerability, RCCEP should be proactively managed rather than considered a contraindication for camrelizumab‐based therapy. In this study, most RCCEP cases were mild and did not require treatment interruption or specific medical intervention, supporting a predominantly conservative management approach. Actually, most RCCEP cases resolve spontaneously after discontinuing camrelizumab, making careful monitoring a priority over aggressive treatment, except in high‐risk bleeding cases where local excision is advisable [35]. While no apparent detrimental impact of RCCEP or its management on clinical outcomes was observed in this analysis, this finding should be interpreted with caution, and further studies are needed to better characterize its potential influence on long‐term prognosis. Future research should focus on refining predictive models, improving anti‐angiogenic strategies [36], and evaluating prophylactic interventions to optimize RCCEP management while balancing oncological efficacy and patient quality of life.

This study has several limitations. First, both the ESCORT and ESCORT‐1st trials were conducted exclusively in Chinese populations, which may limit the generalizability of our findings to other ethnicities and geographic regions. Therefore, external validation in more diverse, multi‐regional cohorts is needed. Second, although comprehensive landmark analyses were employed to reduce immortal time bias, unmeasured confounding variables may still have influenced the outcomes. In particular, other irAEs, which were more frequently observed in patients with RCCEP, may also be associated with improved outcomes and could partially confound the observed associations. More analytical approaches, including time‐dependent modeling, are warranted in future studies to better disentangle the independent effects of RCCEP and other irAEs on clinical outcomes. Conducting sensitivity analyses at multiple landmark time points could further enhance the robustness of the findings. Third, while patients in our analysis were categorized simply as with or without RCCEP, its heterogeneity was not fully explored. The potential impact of RCCEP characteristics, including severity, lesion number, and anatomical distribution, on treatment efficacy warrants further investigation. Fourth, as noted above, the absence of integrated biomarker analyses limits mechanistic interpretation and highlights the need for future studies incorporating molecular and circulating markers.

## 4 Conclusion

In conclusion, this pooled analysis of phase 3 clinical trial data provided robust evidence that RCCEP, a distinctive irAE associated with camrelizumab, was highly prevalent in patients with ESCC and was strongly associated with improved outcomes from camrelizumab‐based therapy. Although patients derived clinical benefit from camrelizumab, either as monotherapy or in combination with chemotherapy, the development of RCCEP was associated with more favorable treatment efficacy. These findings support the potential of RCCEP as an on‐treatment biomarker and highlight the need for further mechanistic and interventional studies to optimize its application in camrelizumab‐based immunotherapy.

## 5 Materials and Methods

### 5.1 Study Design and Patients

This pooled analysis integrated data from two phase III, multicenter, randomized controlled trials, ESCORT (NCT03099382) and ESCORT‐1st (NCT03691090), which evaluated the efficacy and safety of camrelizumab‐based therapies in patients with advanced or metastatic ESCC. Both studies were conducted in accordance with the Declaration of Helsinki and the Good Clinical Practice Guideline and were approved by institutional review boards or independent ethics committees at each participating site. All patients provided written informed consent before enrollment.

Eligible patients were 18–75 years old, had histologically or cytologically confirmed ESCC, and had an Eastern Cooperative Oncology Group (ECOG) performance status of 0 or 1. In the ESCORT trial, patients had progressed on or were intolerant to first‐line chemotherapy, while in ESCORT‐1st, patients had previously untreated, locally advanced, recurrent, or metastatic ESCC. Key inclusion criteria for both trials required patients to have at least one measurable lesion, a life expectancy of at least 12 weeks, and adequate organ function. Exclusion criteria included the presence of other malignancies, active autoimmune disease, symptomatic brain metastases, prior exposure to anti‐PD‐1 or anti‐PD‐L1 therapies, uncontrolled infections, or recent administration of immunosuppressive drugs or live vaccines.

### 5.2 Treatment and Assessments

In ESCORT, patients were randomized 1:1 to receive either camrelizumab (200 mg intravenously every 2 weeks) or chemotherapy with docetaxel (75 mg/m^2^ every 3 weeks) or irinotecan (180 mg/m^2^ every 2 weeks), until disease progression, unacceptable toxicity, patient withdrawal, or investigator decision. In ESCORT‐1st, patients were randomized 1:1 to receive camrelizumab (200 mg intravenously every 3 weeks) or placebo, in combination with up to six cycles of paclitaxel (175 mg/m^2^) and cisplatin (75 mg/m^2^).

Tumor response was evaluated every 6–8 weeks according to the Response Evaluation Criteria in Solid Tumors (RECIST) version 1.1, by both independent review committees and investigators. Patients with radiographic disease progression but clinically stable conditions were allowed to continue treatment until progression was confirmed by a subsequent imaging assessment at least 4 weeks later. ORR was defined as the percentage of patients with complete or partial response. All patients were followed monthly for survival assessment, including PFS (defined as the time from randomization to the first disease progression or death from any cause), and OS (defined as the time from randomization to death from any cause).

### 5.3 RCCEP Grade and Evaluation

The grading criteria for RCCEP were defined as follows: grade 1, nodule(s) ≤ 10 mm, with or without rupture/bleeding; grade 2, nodule(s) with a maximum diameter of > 10 mm, with or without rupture/bleeding; grade 3, generalized nodules throughout the body, possibly with skin infection; grade 4, multiple, generalized nodules, life‐threatening condition; grade 5, death. RCCEP incidence, time to onset, and duration were closely observed and recorded [14].

### 5.4 Statistical Analyses

Demographic and baseline characteristics were summarized for patients who underwent randomization and received at least one dose of camrelizumab‐based therapy (either monotherapy or combination with chemotherapy). ORR, PFS, and OS were evaluated in patients who experienced RCCEP and those who did not. Landmark analyses were conducted to mitigate lead‐time and time‐dependent biases associated with RCCEP occurrence. A 2‐month cutoff was selected based on the median time to first RCCEP onset. Only patients who were alive and progression‐free post the landmark time point were included, and survival outcomes were compared according to RCCEP status [37]. To reduce potential confounding effects from baseline imbalances, inverse probability of treatment weighting (IPTW) was applied. For IPTW, stabilized weights (defined as the weight multiplied by the probability of RCCEP occurrence or not) were calculated from the propensity score and used as weights [38]. Propensity scores were derived through logistic regression, incorporating ECOG performance status (0 vs. 1) and baseline liver metastases (absent vs. present) as covariates. These variables were selected based on their established prognostic relevance in advanced ESCC and the presence of meaningful baseline imbalance (absolute standardized mean differences [SMDs] ≥ 0.1 before IPTW adjustment). SMDs for covariates were calculated before and after weighting to assess balance between groups. In both unadjusted and IPTW‐adjusted analyses, the two‐sided 95% confidence interval (CI) for ORR was calculated using the Clopper–Pearson method. The unadjusted and IPTW‐adjusted median PFS and OS were estimated using the Kaplan–Meier method, with corresponding 95% CIs calculated using the Brookmeyer–Crowley method. Comparisons between groups were performed using the log‐rank test. To evaluate the association between RCCEP occurrence and survival outcomes, unadjusted and IPTW‐adjusted hazard ratios (HRs) with 95% CIs for OS and PFS, were estimated using Cox proportional‐hazards models. The effective sample size (ESS) was used to represent sample size post‐IPTW.

## Author Contributions

Concept and design: Ruihua Xu, Huiyan Luo, and Zhiguo Hou. Acquisition, analysis, or interpretation of data: Huiyan Luo, Jia Liu, Ye He, Mingming He, and Fan Chen. Drafting of the manuscript: Zhaoqing Zheng. Statistical analysis: Chaoyu Lin. Revision of the manuscript: Aichun Chen. All authors read and approved the final manuscript.

## Funding

Both ESCORT and ESCORT‐1st studies included in this pooled analysis were funded by Jiangsu Hengrui Pharmaceuticals Co., Ltd. The sponsor participated in the study design, pooled data analysis, and writing of the report but had no role in data interpretation or the decision to submit the manuscript for publication.

## Ethics Statement

This pooled post hoc analysis was based on data from the ESCORT (NCT03099382) and ESCORT‐1st (NCT03691090) clinical trials. Both studies were approved by the institutional review boards or independent ethics committees at all participating centers. Written informed consent was obtained from all patients prior to enrollment. All data used in the present analysis were de‐identified, and the original informed consent documents permitted secondary analyses of trial data. Both trials were conducted in accordance with the principles of the Declaration of Helsinki and the International Council for Harmonisation Good Clinical Practice (ICH‐GCP) guidelines.

## Conflicts of Interest

Zhiguo Hou, Zhaoqing Zheng, Chaoyu Lin, and Aichun Chen are employees Jiangsu Hengrui Pharmaceuticals Co., Ltd. All other authors declare no conflicts of interest.

## Supporting information




**Supporting Files 1**: mco270917‐sup‐0001‐SuppMat.docx

## Data Availability

The data used and/or analyzed during the present study are included in this published article. Further inquiries can be directed to the corresponding author.
