# Supplementary material for: Reactive Cutaneous Capillary Endothelial Proliferation in Esophageal Squamous Cell Carcinoma Patients Treated With Camrelizumab‐Based Therapy: A Pooled Analysis of Two Phase 3 Trials
Source: MedComm (2020). 2026 Aug 9;7(8):e70917. doi: 10.1002/mco2.70917 (PMC13454563; doi:10.1002/mco2.70917)
Supplement: Supplementary file 1 [file MCO2-7-e70917-s001.docx]

**Title page**

**Reactive cutaneous capillary endothelial proliferation in esophageal squamous cell carcinoma patients treated with camrelizumab-based therapy: a pooled analysis of two phase 3 trials**

Jia Liu^1*^, Ye He^1*^, Mingming He^1^, Fan Chen^1^, Zhiguo Hou^2^, Zhaoqing Zheng^2^, Chaoyu Lin^2^, Aichun Chen^2^, Ruihua Xu^1†^, Huiyan Luo^1†^, ESCORT-1st Study Group, ESCORT Study Group

**Running title：**RCCEP and outcomes in camrelizumab ESCC

* These authors contributed equally to this article.

† Corresponding author.

^1^Department of Medical Oncology, Sun Yat-sen University Cancer Center, Guangzhou 510060, China

^2^ Department of Medical Affairs, Jiangsu Hengrui Pharmaceuticals Co., Ltd., Jiangsu 222047, China

**Corresponding Author:**

Huiyan Luo, MD, PhD, Department of Medical Oncology, Sun Yat-sen University Cancer Center, Guangzhou 510060, China

Email: luohy@sysucc.org.cn

Ruihua Xu, MD, PhD, Department of Medical Oncology, Sun Yat-sen University Cancer Center, Guangzhou 510060, China

Email: [xurh@sysucc.org.cn](mailto:xurh@sysucc.org.cn)

**Supplementary Information**

**Supplementary table S1. PFS and OS for patients with different treatment lines before and after IPTW**

|  | Treatment line | First line (N=298) | | Second line (N=228) | |
| --- | --- | --- | --- | --- | --- |
|  | Before IPTW | With RCCEP  (N=238) | Without RCCEP  (N=60) | With RCCEP  (N=182) | Without RCCEP  (N=46) |
|  | Median PFS, months (95% CI) | 7.16  (6.90-8.25) | 4.40  (2.92-5.82) | 2.79  (1.97-3.68) | 1.12  (0.92-1.51) |
|  | Hazard ratio (95% CI) | 0.49 (0.34-0.70) | | 0.18 (0.12-0.26) | |
|  | Log-Rank test | p < 0.001 | | p < 0.001 | |
|  | Median OS, months (95% CI) | 17.41  (15.05-20.44) | 10.18  (6.31-14.46) | 10.05  (8.54-11.53) | 2.53  (1.91-3.25) |
|  | Hazard ratio (95% CI) | 0.50 (0.36-0.69) | | 0.23 (0.16-0.32) | |
|  | Log-Rank test | p < 0.001 | | p < 0.001 | |
|  | After IPTW | With RCCEP  (N=238.5) | Without RCCEP  (N=62.1) | With RCCEP  (N=181.7) | Without RCCEP  (N=43.1) |
| Median PFS, months (95% CI) | | 7.16  (6.90-8.25) | 5.49  (2.92-6.74) | 2.73  (1.94-3.68) | 1.05  (0.92-1.51) |
| Hazard ratio (95% CI) | | 0.55 (0.38-0.79) | | 0.18 (0.13-0.27) | |
| Log-Rank test | | p = 0.002 | | p < 0.001 | |
| Median OS, months (95% CI) | | 17.41  (15.05-20.44) | 10.68  (6.34-15.64) | 9.99  (8.44-11.40) | 3.06  (1.94-4.40) |
| Hazard ratio (95% CI) | | 0.54 (0.39-0.75) | | 0.27 (0.19-0.38) | |
| Log-Rank test | | p < 0.001 | | p < 0.001 | |

PFS, progression-free survival; OS, overall survival; RCCEP, reactive cutaneous capillary endothelial proliferation; IPTW, inverse probability of treatment weighting

**Supplementary Figure S1**

**Pooled cohort receiving camrelizumab-based therapy**

**(N = 526)**

**501 included in OS landmark analysis**

**With RCCEP / Without RCCEP	Disease progression (n = 84 / n = 36)**

**New anti-tumor therapy initiated**

**(n = 4 / n = 8)**

**Death (n = 4 / n = 14)**

**Insufficient tumor assessments**

**(n = 1 / n = 6)**

**OS Landmark Analysis**

**PFS Landmark Analysis**

**With RCCEP / Without RCCEP**

**Death (n = 4 / n = 21)**

**369 included in PFS landmark analysis**

**Supplementary Figure S1. Flow chart of landmark analysis populations**

Patients who experienced progression, death, initiation of new antitumor therapy, or inadequate tumor assessment before the 2-month landmark time point were excluded from the landmark analyses.
